# Distinguishing the Roles of the Dorsomedial Prefrontal Cortex and Right Temporoparietal Junction in Altruism in Situations of Inequality: A Transcranial Direct Current Stimulation Study

**DOI:** 10.3389/fnhum.2022.821360

**Published:** 2022-03-23

**Authors:** Hanqi Zhang, Zhiqiang Dong, Shenggang Cai, Jun Zhao

**Affiliations:** ^1^Key Lab for Behavioral Economic Science and Technology, South China Normal University, Guangzhou, China; ^2^School of Economics and Management, South China Normal University, Guangzhou, China

**Keywords:** altruism, inequality, dorsomedial prefrontal cortex, right temporoparietal junction, transcranial direct current stimulation

## Abstract

The right temporoparietal junction (rTPJ) and dorsomedial prefrontal cortex (dmPFC), which are involved in social cognition, have been proposed to play key roles in guiding human altruistic behavior. However, no study has provided empirical evidence that the rTPJ and dmPFC play distinct roles in altruism under situations of inequality. A total of 107 healthy young adults were randomly assigned to receive anodal or sham transcranial direct current stimulation (tDCS) to either the dmPFC or rTPJ, and they participated in a modified dictator game. The stimulation of the dmPFC increased the level of altruistic behavior, while the stimulation of the rTPJ did not. Furthermore, we determined that the increase in altruism induced by tDCS of the dmPFC could be modulated by perspective taking. These results demonstrate that the dmPFC and rTPJ play distinct roles in the enhancement of altruism in situations of inequality; this finding is consistent with theories proposing that the dmPFC has evolved mechanisms dedicated to perspective taking.

## Introduction

Altruistic acts benefit others at some cost to oneself (Fehr and Fischbacher, 2003; Morishima et al., 2012). From a biological or evolutionary perspective, altruism decreases the fitness or genetic contribution of one individual while increasing the fitness of another (Filkowski et al., 2016). For the individual, altruistic behavior reduces one’s chances of survival and reproduction. However, for society, altruistic acts facilitate human cooperation (Fehr and Fischbacher, 2003; Wang et al., 2020) and enable members of a group to survive (Tomasello, 2009).

Altruism is one of the major puzzles in the behavioral sciences today (Hardy and Van Vugt, 2006) and is subject to intense interest from psychologists and evolutionary biologists. The drivers of human altruism constitute a highly enduring and contentious question in psychology (Waytz et al., 2012). Many key evolutionary theories of altruism have emerged to explain human altruism, such as kin selection theory (Hamilton, 1964), reciprocal altruism theory (Trivers, 1971), and competitive altruism theory (Hardy and Van Vugt, 2006). Altruism is thought to rely, in part, from the ability to understand other mental states (Waytz et al., 2012). Altruistic behaviors are likely to be related to humanity’s unique capacity for mentalizing, or reflecting on and understanding the minds of others (Morishima et al., 2012).

One of the most consistent viewpoints in cognitive neuroscience is that the medial prefrontal cortex (mPFC, especially the dorsal mPFC) and right temporoparietal junction (rTPJ) comprise the mentalizing network (Van Overwalle and Baetens, 2009; Waytz et al., 2012). The mPFC (Hu et al., 2017) and rTPJ (Tusche et al., 2016) have also been proposed to play key roles in guiding human altruistic behavior. Researchers have discovered that the response of the dmPFC predicts altruistic behavior (Waytz et al., 2012) and that the anodal transcranial direct current stimulation (tDCS) over the mPFC increases an individual’s propensity for altruism comparing with the cathodal tDCS (Liao et al., 2018). Furthermore, altruism is correlated with gray matter volume in the rTPJ (Morishima et al., 2012). However, a transcranial magnetic stimulation (TMS) study by Obeso et al. (2018) demonstrated that the rTPJ governs moral conflicts related to altruistic behavior. Furthermore, a tDCS study by Martin et al. (2019) determined that the dmPFC and rTPJ have dissociable roles in self–other processing, which plays a key role in altruism. These studies provide causal evidence for the role of the rTPJ and dmPFC related to social, moral, and altruistic behavior, but they have not specifically measured the distinct role between dmPFC and rTPJ in altruism in situation of inequity.

Research on human altruism has provided evidence that altruistic behaviors and motives depend on the payoff allocation between the actor and the recipient of the act (Tricomi et al., 2010; Morishima et al., 2012). The situation of inequity might change actor’s moral conflict owning to the different advantages between the actor and the recipient of the act. The propensity toward behaving altruistically differs in situations of advantageous vs. disadvantageous inequality (Tricomi et al., 2010), the neuroanatomical basis for human altruism might be dissimilar between the two domains. A voxel-based morphometric study determined that altruistic behaviors in the domain of advantageous inequality are correlated with gray matter volume in the rTPJ (Morishima et al., 2012), whereas altruistic acts in domain of disadvantageous inequality are correlated with gray matter volume in the mPFC. These results indicate that the dmPFC and rTPJ may play different roles in altruism in different domains of inequality. However, no study has provided empirical evidence that the dmPFC and rTPJ play distinct roles in altruistic behaviors under situations of inequality.

In this study, we explored the causal roles of the dmPFC and rTPJ in altruistic acts in situations of inequality by applying transcranial direct current stimulation (tDCS) to 107 participants who were confronted with a series of binary choice problems related to the allocation of money between themselves and an anonymous partner. The tDCS is a non-invasive procedure that applies weak electric currents to the scalp to modulate neural excitability in healthy humans (Nitsche and Paulus, 2011; Woods et al., 2016; Maréchal et al., 2017; Meyer et al., 2019; Wang and Zhang, 2021; Xiong et al., 2021). The mechanism of tDCS is likely to revolve around a slight modulation of the membrane potentials and the spontaneous firing rate of the stimulated neurons (Stagg et al., 2018; Meyer et al., 2019), or the induction of plastic after-effects via alteration of neurotransmitter activity (Stagg et al., 2018). Anodal stimulation is thought to increase neural excitability, whereas cathodal stimulation is thought to decrease neural excitability.

In our study, we administered (1) anodal tDCS to the dmPFC (*n* = 36, with 12 men) or rTPJ (*n* = 35, with 17 men) to exogenously enhance neural excitability and (2) sham tDCS to the dmPFC or rTPJ (*n* = 33, with 10 men) of each participant in the control group. Our study empirically uncovered the neural mechanisms that regulate altruistic behavior in situations of inequality: we found that anodal stimulation of the dmPFC increased the level of altruistic behavior but anodal stimulation of the rTPJ did not affect the level of altruistic behavior.

## Materials and Methods

### Experimental Design

This study employed a single-blind, sham-controlled, between-subjects design. The participants randomly underwent one of the three types of stimulation: (1) anodal stimulation of the dmPFC, (2) anodal stimulation of the rTPJ, or (3) sham stimulation of the dmPFC or rTPJ.

### Subjects

We used G*Power to compute required sample size in need to performed one way ANOVA analysis, the required sample size is 102. In total, 107 healthy individuals from South China Normal University participated in this study (68 women and 39 men, mean age = 20.07 ± 1.55 years) and received a monetary award for their time. All the participants provided written informed consent in accordance with procedures approved by the South China Normal University Ethics Committee. We excluded the data of three participants who failed five times to complete the required questionnaires.

### Questionnaires

After completing the modified dictator game task, the participants were instructed to complete questions based on two subscales of the Interpersonal Reactivity Index (IRI) and the Dispositional Greed Scale. The first subscale of the IRI used was the perspective taking subscale, which measures participants’ tendency to spontaneously adopt the views of someone else and how much the participants put themselves in the shoes of someone else; the other subscale of IRI used was the empathic concern subscale, which assesses participants’ tendency to experience sympathy and compassion for unfortunate others (Wu et al., 2021). The 7-item Dispositional Greed Scale was used to measure the level of greed (Li et al., 2019).

### Transcranial Direct Current Stimulation

The stimulation was administered using a one-channel direct current stimulator (DC-STIMULATOR; neuroConn, Ilmenau, Germany) and saline-soaked surface sponge electrodes (5 × 7 cm; Xiong et al., 2019). The FPz and Fz electrode sites were first measured and identified for each participant. The scalp region overlying the dmPFC was located by measuring 15% of the distance from the Fz to the FPz (Martin et al., 2019). In accordance with previous studies, the reference electrode was placed over the arm (Xiong et al., 2019). The rTPJ was located at CP6 electrode site according to the international electroencephalography (EEG) 10–20 system.

The current faded in from 0 to 1.5 mA over 30 s. In the treatment group, tDCS was administered to the dmPFC or rTPJ at 1.5 mA for 20 min after the fade-in of the current. In the control group, electrodes were attached to the dmPFC for half of the participants and to the rTPJ for the other half, and the direct current remained at 1.5 mA for 30 s before the fade-out of the current. We used offline tDCS which was dependent on the after-effects and was applied before the task. We chose offline stimulation because previous studies have shown that anodal tDCS enhances and cathodal tDCS reduces cortical excitability in the long term and offline stimulation is more effective than online stimulation (Jacobson et al., 2012; Pirulli et al., 2013; Hao et al., 2021).

### Task and Procedure

**FIGURE 1:**
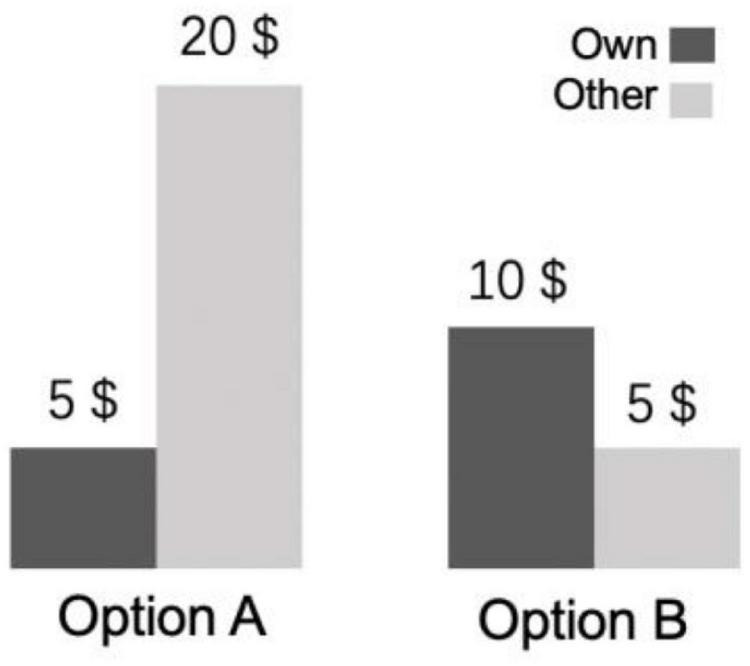
Experimental procedure. Participants faced many decision problems in the dictator game. They had to choose one of two payoff allocations (“options”) that assigned money to the participant (own) and an anonymous other. Subject could make an altruistic choice (i.e., option A) that increases other’s payoff (other gets $15 more, benefit = $15) at a cost to participant (gets $5 less, cost = $5).

We used a dictator game to evaluate the participants’ altruistic propensities in situations of inequality. In the dictator game, the participants were instructed to allocate money to themselves and to an anonymous partner by choosing one of two payoff options (Option A or Option B). One option involved the participants receiving a lower payoff than their partner; the other option involved the participants having a higher payoff than their partner (Figure 1). In this unequal situation, altruism refers to the act that participants sacrifice their self-interest to improve others’ welfare by choosing a lower payoff than their anonymous partners.

The participants were randomly assigned to one of three groups: the rTPJ treatment group, the dmPFC treatment group, or the sham group. After tDCS, the participants were required to complete a modified dictator game, with 10 randomly presented trials based on the parameters stipulated by Zhu et al. (2014). The average payoff of 5 trials was taken as the payment for participants. Finally, the participants were instructed to complete the IRI subscales and Dispositional Greed Scale.

## Results

### Analysis of Means

**FIGURE 2:**
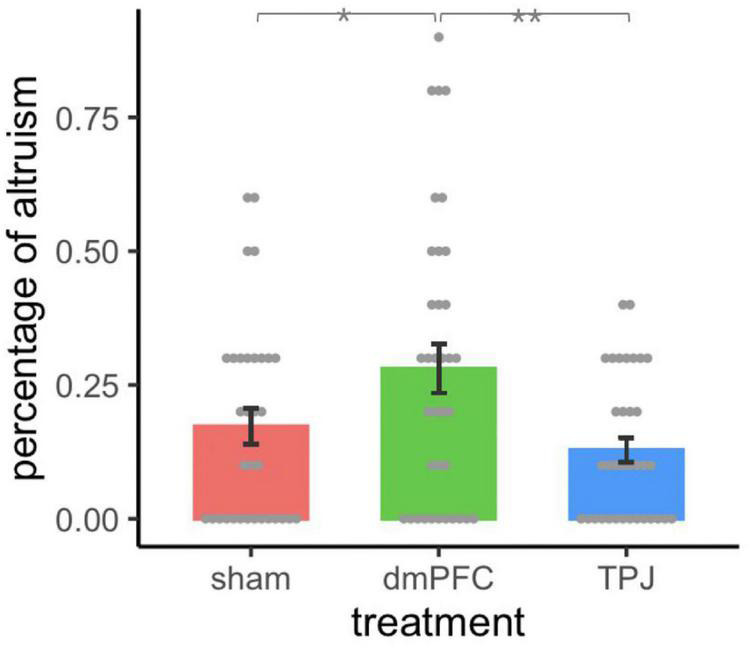
The percentage of altruistic choices among different groups. Participants who underwent tDCS of the dmPFC exhibited a significantly higher percentage of altruism than did those who underwent sham stimulation (*p* = 0.016) or tDCS of the rTPJ (*p* = 0.001), whereas no significant different was identified between the rTPJ experimental group and control group (*p* = 0.377). Error bars indicate ± 1 standard error of mean. Asterisks indicate significance levels: *p < 0.05; **p < 0.01.

We defined altruistic choices as choices in which participants sacrifice their own payoff to improve their partners’ welfare and result in a lower payoff than their partners. As the distributions of percentage of altruism are not normal distribution (Kolmogorov-Smirnov test, sham condition *p* = 0.040; TPJ condition *p* = 0.047, dmPFC condition *p* = 0.288), we used log altruism [−log_10_(2-percentage of altruism), Kolmogorov-Smirnov test, sham condition *p* = 0.053; TPJ condition *p* = 0.055, dmPFC condition *p* = 0.181] as dependent variable to perform one-way ANOVA analysis, observed a significant treatment effect of tDCS on altruistic choices [*F*_(2,__101)_ = 6.143, *p* = 0.003, η^2^ = 0.108]. The results of the *post-hoc* tests indicated that the participants who underwent tDCS of the dmPFC exhibited a significantly higher percentage of altruism than did those who underwent sham stimulation (*p* = 0.016, η^2^ = 0.056) or tDCS of the rTPJ (*p* = 0.001, η^2^ = 0.102). However, no significant difference was identified between participants in the rTPJ treatment group and the control group (*p* = 0.377) (Figure 2). Furthermore, Kruskal-Wallis test also found that treatment effect of tDCS on altruism was significant [χ(2) = 6.57, *p* = 0.037].

As gender effects have been identified in relation to social cognition and tDCS, especially at the mPFC (Martin et al., 2017). We used log altruism as dependent variable to performed 2 (gender:*malevs*.*female*) × 3 (treatment: Sham vs. dmPFC vs. rTPJ) two-way ANOVA analysis. The interaction between treatment and gender was not significant [*F*_(2,__98)_ = 1.936, *p* = 0.150], and the main effect of gender was not significant [*F*(1, 98) = 0.020, *p* = 0.889], while the main effect of treatment was significant [*F*_(2,__98)_ = 4.069, *p* = 0.020, η^2^ = 0.077]. We used the ARTool package in R to perform a non-parametric analysis found similar results [Interaction effect, *F*_(2,__98)_ = 2.08, *p* = 0.130; gender effect, *F*_(1,__98)_ = 0.06, *p* = 0.810; treatment effect, *F*_(2,__98)_ = 3.46, *p* = 0.035].

### Logistic Regression Analysis

To more directly characterize the effects of the stimulation of dmPFC and rTPJ on other-regarding behavior, we examined how the participant’s altruistic acts were influenced by the tDCS treatment, gender, partner’s benefit of altruism and subject’s cost of altruism (benefit is the absolute value of difference in the partner’s payoff between the two options, cost is the absolute value of difference in subject’s payoff between the two options). We used the *lme4* package in R to perform a logistic regression analysis regarding the participants’ decisions, in which the tDCS treatment was a category variable with the sham stimulation as the baseline group. Participants’ choices in each trial were the dependent variable in which the altruistic choice were denoted as 1, otherwise as 0.


P(altruism)=β+0β×1dmFC+β×2TPJ+β×3benefit+β×4cost+β×5gender+β×6dmPFC×benefit+β×7TPJ×benefit+β×8dmPFC×gender+β×9TPJ×gender

As shown in Table 1, the results indicated that participants’ altruistic acts were affected by anodal tDCS over dmPFC. We found that the tDCS of the dmPFC was significant (β = 1.56, *p* = 0.018); in other words, anodal tDCS of the dmPFC increased participants’ altruistic tendency. This result indicates that anodal tDCS of the dmPFC made the participants more other-regarding. However, the treatment effect of tDCS over the rTPJ was non-significant (β = −0.21, *p* = 0.780); in other words, anodal tDCS of the rTPJ did not increase the participants’ altruistic tendency. It indicates that the anodal tDCS of the rTPJ showed little effect on other-regarding propensities in situations of inequality. The interaction between dmPFC and gender was significant (β = −2.20, *p* = 0.028), gender effect was not significant (β = 1.36, *p* = 0.066). Furthermore, we used anodal tDCS stimulation on rTPJ as the baseline group to perform the logistic regression analysis, the treatment effect between dmPFC and rTPJ was significant (β = 1.78, *p* = 0.012, Supplementary Table 2). It indicates that the anodal tDCS of the dmPFC and rTPJ play different role on other-regarding propensities in situations of inequality.

**TABLE 1:** Logistic regression coefficients indicating the effects of tDCS treatment, gender, benefit, and cost of altruism on altruistic behavior.

	Estimate	se	*p*
(Intercept)	–2.48	0.52	0.000***
TPJ	–0.21	0.75	0.780
dmPFC	1.56	0.66	0.018*
Benefit	0.16	0.04	0.000***
Cost	–0.29	0.03	0.000***
Gender	1.36	0.74	0.066
TPJ × benefit	0.02	0.05	0.684
dmPFC × benefit	0.01	0.04	0.747
TPJ × gender	–1.07	1.01	0.291
dmPFC × gender	–2.20	1.00	0.028*

*dmPFC, anodal tDCS of the dmPFC; TPJ, anodal tDCS of the rTPJ.*

*p < 0.05; ***p < 0.001.

### Mechanism

We explored possible mechanisms for why anodal tDCS increased altruistic act. To assess whether anodal tDCS of the dmPFC and rTPJ increased altruism by weakening material self-interest, we administered a greed questionnaire to evaluate how selfish the participants were. The altruism was negatively correlated with greed (Spearman’s rho = −0.431, *p* < 0.001). However, an analysis of ANOVA indicated that tDCS did not affect greed (*p* = 0.824, Supplementary Table 4), and an analysis of ANCOVA indicated that the effect of anodal tDCS on log altruism [*F*_(2,__100)_ = 7.454, *p* = 0.001, η^2^ = 0.130] was not affected as greed scores were controlled. Linear regression analysis found that the greed did not modulate treatment effect on log altruism [dmPFC × greed (β = −0.003, *p* = 0.427) and TPJ × greed (β = 0.002, *p* = 0.582)]. These results suggest that the effect of anodal tDCS of dmPFC on altruistic acts was not due to weakening material self-interest.

To assess whether anodal tDCS increased altruism by strengthening empathy, we used the empathy subscale of the IRI to measure the participants’ empathy. The altruism was not correlated with the participants’ empathy (*p* = 0.082), an analysis of ANOVA indicated that tDCS did not affect empathy (*p* = 0.366, Supplementary Table 4), and also an analysis of ANCOVA indicated that the effect of anodal tDCS on log altruism [*F*_(2,__100)_ = 5.677, *p* = 0.005, η^2^ = 0.102] was not affected as empathy was controlled. Linear regression analysis with sham as baseline group found that the greed did not modulate treatment effect on log altruism [dmPFC × empathy (β = −0.003, *p* = 0.641) and TPJ × empathy (β = −0.001, *p* = 0.850)]. These results suggest that the increase in altruistic acts in situations of inequality caused by anodal tDCS of the dmPFC is not attributable to strengthened empathy.

We then examined whether anodal tDCS of the dmPFC was involved in an increase in perspective taking. We used the perspective taking subscale of the IRI to measure the participants’ perspective taking. An analysis of ANOVA indicated that tDCS did not affect perspective taking (*p* = 0.667, Supplementary Table 4). The percentage of altruistic behavior was not correlated with the participants’ perspective taking (*p* = 0.117), and an analysis of ANCOVA indicated that the effect of anodal tDCS on log altruism [*F*_(2,__100)_ = 6.265, *p* = 0.003, η^2^ = 0.111] was affected as perspective was controlled. However, Linear regression analysis indicated that the interaction between dmPFC × perspective taking was significant (β = −0.01, *p* = 0.04, Table 2). Subjects’ perspective taking modulated the treatment effect on log altruism; the lower perspective taking, the stronger tDCS effect. This result suggests that the altruistic acts in situations of inequality caused by anodal tDCS might be modulated by perspective taking.

**TABLE 2:** Regression analysis indicating the effects of tDCS treatment, and perspective taking on log altruism.

Coefficient	Estimate	Se	*p*
(Intercept)	−0.26	0.05	0.00
dmPFC	0.18	0.07	0.01*
TPJ	0.01	0.07	0.86
PT	0.00	0.00	0.93
dmPFC × PT	−0.01	0.00	0.04*
TPJ × PT	0.00	0.00	0.74

*dmPFC, anodal tDCS of the dmPFC; TPJ, anodal tDCS of the rTPJ; PT, perspective taking.*

*p < 0.05.

## Discussion

This study used a modified dictator game to demonstrate that dmPFC and rTPJ play distinct roles in altruism in situations of inequality. Anodal tDCS of the rTPJ did not affect altruistic behavior in the situations of inequality; however, anodal tDCS of the dmPFC increased altruistic behavior in situations of inequality. Furthermore, this study found that the altruistic acts in situations of inequality caused by anodal tDCS might be modulated by perspective taking.

Perspective taking involves going beyond one’s own perspective to consider a given situation from another party’s perspective (Ku et al., 2015; Ortiz-Riomalo et al., 2021), is. Scholars noted that individuals who have a tendency to actively engage in perspective taking—understand other people’s thoughts and feelings, are motivated to perform altruistically (Oswald, 1996; Li, 2011). Cárdenas (2018) determined that individuals make choices based not only on self-regarding but also on other-regarding preferences. Individuals holding other-regarding preferences may act in favor of others whose perspectives they are considering. A behavioral study by Ortiz-Riomalo et al. (2021) reported that inducing perspective taking promotes altruistic behavior. A tDCS study by Martin et al. (2019) reported that the dmPFC is involved in self-other processing and the high-definition tDCS of the dmPFC increases the influence of allocentric perspectives. Schäfer and Frings (2019) found that tDCS over mPFC did not affect self-prioritization effect. With respect to electrode size, electrode position, current strength and stimulation duration, the employed tDCS procedures show strong variations (Friehs et al., 2021). Compared to the study of Schäfer and Frings (2019) applying stimulation to the ventral parts of mPFC, the present study and the study by Martin et al. (2019) applied stimulation to dorsal part of mPFC. Neural data revealed a spatial gradient in value representation along the mPFC involving prosociality (Sul et al., 2015).

Our study elucidates the distinct roles played by the dmPFC and rTPJ in altruism. The dmPFC and rTPJ have been proposed to play key roles in guiding human altruistic behavior. One of the most consistent viewpoints in cognitive neuroscience is that the mPFC and the rTPJ comprise the mentalizing network (Van Overwalle and Baetens, 2009; Waytz et al., 2012). Neuroimaging and neurostimulation studies have suggested the involvement of the dmPFC in altruistic behaviors. Researchers have determined that dmPFC response is associated with altruistic behavior (Waytz et al., 2012) and that anodal tDCS stimulation of the mPFC increases an individual’s propensity for altruism (Liao et al., 2018). These findings are consistent with those of our study, which also demonstrates that the dmPFC plays a key role in mentalization (i.e., perspective taking). However, our study did not demonstrate a causal relationship between the cortical excitability of the rTPJ and altruism in situations of inequality. This finding is consistent with the tDCS findings of Blair-West et al. (2018), who observed that the rTPJ was not involved in social decision-making when participants were playing an ultimatum game.

The present study investigated the causal role of the dmPFC in enhancing altruism in situations of inequality. Altruistic behaviors and motives depend on the initial allocation of resources (Tricomi et al., 2010; Morishima et al., 2012), but the distinct roles played by the dmPFC and rTPJ in altruism under situations of inequality have remained unclear. A TMS study by Obeso et al. (2018) reported that the rTPJ governs the processing of moral conflicts related to altruistic behavior. In the present study, anodal tDCS of the rTPJ did not increase altruistic behavior in situations of inequality. A possible reason is that altruistic behavior in such situation does not involve the perception of moral conflict. In situations of choosing between advantage inequality and an equal option, choosing the equal option is often prescribed by an individual’s moral motives. However, choosing between disadvantage inequality and an equal option is often not involved in moral motives due to the lower payoff of the participant in the disadvantage option. In the present study, the choice of the disadvantage option is indicated as altruism, which is similar to in situations of disadvantage inequality. Thus, we can infer that the anodal tDCS of the rTPJ did not affect the altruistic act in situations of our present study by modulating moral conflict.

This study has several limitations. First, we did not distinguish between disadvantageous inequality (i.e., participants receiving a higher payoff than their partner) and advantageous inequality (i.e., participants receiving lower payoff than their partner) in our experiments. Rather, we employed mixed inequality: one option for disadvantageous initial inequality and another for advantageous initial inequality. A voxel-based morphometric study reported that altruism in situations of disadvantageous inequality was not correlated with altruism in situations of advantageous inequality; furthermore, gray matter volume in the rTPJ is strongly associated with individuals’ altruism in situations of advantageous inequality but not situations of disadvantageous inequality (Morishima et al., 2012). In our study, to exhibit altruism, the participants had to choose a disadvantageous option. This altruistic decision might be more similar to the altruistic decision in a disadvantageous inequality situation than to one in an advantageous inequality situation. The lack of effect exerted by anodal tDCS of the rTPJ on altruistic behavior in this study is consistent with the findings of previous studies. Therefore, the rTPJ may play a distinct role in advantageous inequality situations. Additional studies should distinguish these types of initial inequalities.

Future studies should employ tDCS to dissociate the neural mechanism on altruism in advantageous situations and in disadvantageous situations. Research on altruism has demonstrated no correlation between an individual’s propensity for behaving altruistically in situations of advantageous inequality and in those of disadvantageous inequality. The neuroanatomical basis for human altruism is also dissimilar across these domains. Studies have reported that altruistic preferences under situations of advantageous inequality are correlated with gray matter volume in the rTPJ but not with that in the dmPFC (Morishima et al., 2012). However, our study demonstrates the causal relationship between altruism under the situations of inequality and the cortical excitability of the dmPFC but not of the rTPJ. In this study, the participants exhibited their altruism by choosing a disadvantage option, which is similar to altruism in the situations of disadvantage inequality. Therefore, altruism in advantageous situations may be dissociable from altruism in disadvantageous situations.

## Data Availability Statement

The raw data supporting the conclusions of this article will be made available by the authors, without undue reservation.

## Ethics Statement

The studies involving human participants were reviewed and approved by the Ethics Committee of South China Normal University. The patients/participants provided their written informed consent to participate in this study.

## Author Contributions

HZ, ZD, SC, and JZ participated in the design of this study, carried out the study, constructed the overall framework of the study, and modified and polished it. HZ and JZ performed the statistical analysis, collected important background information, and drafted the manuscript. All authors read and approved the final manuscript.

## Conflict of Interest

The authors declare that the research was conducted in the absence of any commercial or financial relationships that could be construed as a potential conflict of interest.

## Publisher’s Note

All claims expressed in this article are solely those of the authors and do not necessarily represent those of their affiliated organizations, or those of the publisher, the editors and the reviewers. Any product that may be evaluated in this article, or claim that may be made by its manufacturer, is not guaranteed or endorsed by the publisher.
