# Supplementary material for: Distinguishing the Roles of the Dorsomedial Prefrontal Cortex and Right Temporoparietal Junction in Altruism in Situations of Inequality: A Transcranial Direct Current Stimulation Study
Source: Front Hum Neurosci. 2022 Mar 23;16:821360. doi: 10.3389/fnhum.2022.821360 (PMC8985852; doi:10.3389/fnhum.2022.821360)
Supplement: Supplementary file 1 [file Data_Sheet_1.pdf]

Supplementary Material

Table s1. full table of options

Option A		Option B	
**own**	**other**	**own**	**other**
12	10	10	12
5	15	6	5
10	4.99	4	5
15	5	5	15
10	5	5	10
6	5	5	10
10	4.99	6	5
5	20	10	5
6	5	5	6
5	20	20	5

Table s2. Logistic regression coefficients indicating the effects of tDCS treatment, gender, benefit and cost of altruism on altruistic behavior (TPJ as baseline).

	Estimate	se	p
(Intercept)	-2.70	0.57	0.000
Sham	0.22	0.75	0.772
dmPFC	1.78	0.71	0.012
benefit	0.18	0.04	0.000
cost	-0.29	0.03	0.000
gender	0.28	0.69	0.690
sham×benefit	-0.02	0.05	0.635
dmPFC×benefit	-0.01	0.05	0.865
sham×gender	1.09	1.01	0.282
dmPFC×gender	-1.13	0.97	0.246

Table s3. Logistic regression coefficients indicating the effects of tDCS treatment, efficiency, and gender on altruistic behavior.

Coefficient	Estimate	se	p
(Intercept)	-3.54	0.51	0.000
dmPFC	1.68	0.62	0.007 **
TPJ	-0.08	0.71	0.910
efficiency	0.29	0.05	0.000 ***
gender	1.36	0.72	0.060
dmPFC × efficiency	-0.01	0.07	0.843
TPJ × efficiency	0.01	0.07	0.925
dmPFC× gender	-2.16	0.98	0.028 *
TPJ × gender	-1.07	0.99	0.282

efficiency = benefit / cost

Table s4. Effect of tDCS on greed, empathy, and perspective taking.

Stimulation group	Statistics	Greed	Empathy	Perspective Taking
Sham(n=33)	Mean	23.76	17.79	17.82
	sd	4.41	3.14	3.66
dmPFC(n=36)	Mean	23.39	18.75	18.33
	sd	3.24	3.14	3.11
TPJ(n=35)	Mean	23.11	18.11	18.51
	sd	4.99	2.69	3.11
F-test	p value	0.824	0.366	0.667
